# Associations between skeletal muscle phenotype, positional role, and on‐ice performance in elite male ice hockey players

**DOI:** 10.14814/phy2.70081

**Published:** 2024-11-10

**Authors:** Jeppe F. Vigh‐Larsen, Hallur Thorsteinsson, Martin Thomassen, Jeppe Panduro, Bjørn Fristrup, Morten B. Randers, Jens L. Olesen, Peter Krustrup, Kristian Overgaard, Lars Nybo, Magni Mohr

**Affiliations:** ^1^ Department of Sports Science and Clinical Biomechanics, SDU Sport and Health Sciences Cluster (SHSC) University of Southern Denmark Odense Denmark; ^2^ Department of Nutrition, Exercise and Sports University of Copenhagen Copenhagen Denmark; ^3^ Department of Clinical Medicine, The Faculty of Medicine Aalborg University Aalborg Denmark; ^4^ Danish Institute for Advanced Study (DIAS) University of Southern Denmark Odense Denmark; ^5^ Sport and Health Sciences University of Exeter Exeter UK; ^6^ Department of Public Health, Section of Sport Science Aarhus University Aarhus Denmark; ^7^ Centre of Health Science, Faculty of Health University of the Faroe Islands Tórshavn Faroe Islands

**Keywords:** fatigue, high intensity intermittent exercise, ion transporters, team sports, testing, tracking

## Abstract

We evaluated associations between muscle phenotype, positional role, and on‐ice performance in male U20 Danish national team ice hockey players. Sixteen players (10 forwards, six defensemen) participated in a game with activity tracking. Resting thigh muscle biopsies were analyzed for metabolic enzyme activity and protein expression linked to performance. On‐ice intermittent exercise capacity, repeated sprint ability, and maximal isometric knee‐extensor torque were also assessed. No significant position‐specific muscle phenotype characteristics were found, but forwards generally exhibited higher levels of several membrane proteins (*p* = 0.100–0.991). NAKα_2_, NAK∑, K_ATP_, ClC‐1, and NHE1 showed significant correlations with total distance (*r* = 0.52–0.59, *p* = 0.016–0.046), however, within positions these only persisted for K_ATP_ (*r* = 0.70, *p* = 0.024) and NAKα_2_ (*r* = 0.57, *p* = 0.085) in forwards, where CS enzyme activity also displayed a strong association with distance covered (*r* = 0.75, *p* = 0.019). For high‐intensity skating, NAKα_2_ (*r* = 0.56, *p* = 0.025) and K_ATP_ (*r* = 0.50, *p* = 0.048) similarly exhibited the strongest associations, persisting within forwards (*r* = 0.63, *p* = 0.052 and *r* = 0.72; *p* = 0.018, respectively). In conclusion, although several muscle proteins involved in ion and metabolic regulation were associated with performance, only NAKα_2_ and K_ATP_ displayed consistent relationships within positions. Moreover, CS enzyme activity was strongly related to total distance within forwards, coherent with the proposed importance of oxidative capacity in intense intermittent exercise.

## Graphical abstract

Protein expression of NAKα_2_ and K_ATP_ was most consistently associated with total and high‐intensity distance covered per min, while citrate synthase enzyme activity was strongly associated with total distance covered per min in forwards. Although overall correlations were present for other proteins, these were not consistent within different positional roles.

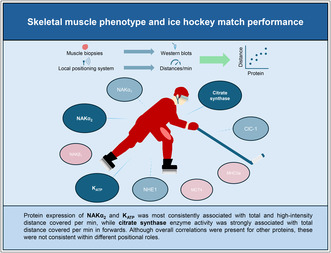

## 1 INTRODUCTION

Skeletal muscles that are intensively activated for a period of time display a gradual but transient decline in force output, commonly known as fatigue (Allen et al., 2008). While the underlying physiological components important for continuous endurance exercise performance are well described (Joyner & Coyle, 2008), the physiological underpinnings of performance in intermittent sports are less well understood. This is due to the high complexity and hybrid nature of intermittent exercise, taxing both aerobic and anaerobic energy systems and processes of excitation‐contraction coupling in exercise scenarios where different types of fatigue may occur (Cairns & Lindinger, 2008; McKenna et al., 1985). Thus, performance and exercise tolerance are likely to depend on an interplay between a myriad of physiological systems.

During high intensity exercise the membrane excitability may be compromised due to large transmembrane ion shifts and potential reductions in cellular energy homeostasis (Hostrup et al., 2021; Renaud et al., 2023). Also, disruptions in intracellular metabolites may affect several key steps in the excitation‐contraction coupling, resulting in deteriorated muscle force production (Hostrup & Bangsbo, 2017). For example, during high intensity exercise K^+^ is released from the muscle cell through voltage gated K^+^ channels causing an extracellular accumulation of K^+^, which, if sufficiently severe, may reduce sarcolemmal excitability (McKenna et al., 1985; Nordsborg et al., 2003; Overgaard & Nielsen, 2001). In contrast, moderate elevations of extracellular K^+^ may potentiate muscle performance (Olesen et al., 2021; Overgaard et al., 2022; Pedersen et al., 2019), indicating that balancing the transport rates of K^+^ across the sarcolemmal membrane is crucial to muscle function. In addition, during exhaustive exercise muscle lactate accumulates within the muscle cell and intramuscular pH is reduced which may negatively affect performance as indicated in human (Juel et al., 1990) and rodent muscle (Knuth et al., 2006), although beneficial effects of increased acidification have also been observed in vitro (Nielsen et al., 2001; Overgaard et al., 2010).

Muscle cells have numerous membrane proteins functioning as transporters, exchangers and channels that counteract ionic and metabolic shifts to preserve intramuscular homeostasis (Allen et al., 2008; Baekgaard Nielsen et al., 2017; Clausen, 2003). Therefore, these proteins are deemed important for maintenance of muscle function and performance during high‐intensity exercise (Hostrup et al., 2021; Hostrup & Bangsbo, 2017). Indeed, several studies have demonstrated that a period of high intense training upregulates the expression of ion handling and metabolite exchange proteins with concomitant improvement in exercise performance (Gunnarsson et al., 2012; Gunnarsson et al., 2013; Skovgaard et al., 2014; Thomassen et al., 1985; Vorup et al., 2016), implying that ion and metabolite handling is important for exercise performance. Therefore, it is interesting to investigate the skeletal muscle phenotype in elite athletes in relation to exercise performance to gain a more thorough understanding of the physiological performance underpinnings, which may ultimately lead to improved training strategies to enhance performance.

Ice hockey is a high‐intense intermittent activity performed over a duration of 60 min of active playing time (Montgomery, 1988; Vigh‐Larsen & Mohr, 2024). In a single game the most utilized players may reach more than 25 min of active time on‐ice and cover more than 6000 m of skating (Brocherie et al., 2018; Douglas & Kennedy, 2020; Gamble et al., 2022; Lignell et al., 2018; Vigh‐Larsen, Ermidis, Rago, et al., 2020). During shifts, the exercise intensity is high and players cover nearly half of the distance in high‐speed skating (>17 km/h), reaching near maximal heart rates and performing frequent explosive actions such as accelerations, decelerations, changes of direction and body checks (Gamble et al., 2022; Lignell et al., 2018; Vigh‐Larsen, Ermidis, Rago, et al., 2020). The hybrid nature of the activity during an ice hockey game requires elite players to possess a well‐rounded physique ranging from a moderate‐to‐high aerobic capacity to a high rate of force development (Green, 1987; Peterson et al., 2016; Sommer Jeppesen et al., 2022; Vigh‐Larsen et al., 2019; Vigh‐Larsen, Haverinen, Knudsen, et al., 2020; Vigh‐Larsen, Haverinen, Panduro, et al., 2020). Thus, a variety of physiological components important for endurance, high‐intensity and explosive exercise performance may interact to determine exercise capacity in an ice hockey game.

In previous studies by Iaia et al. (2008); Iaia et al. (2011), specific muscle phenotype measures were positively associated with intense cycling exercise performance of ~1–4 min duration, as well as with repeated intense running performance, including the relative expression of subunits of NAK, regulating muscle transmembrane Na^+^ and K^+^ gradients, and NHE1 involved in muscle proton efflux. Also, in competitive soccer, skeletal muscle phenotype has been shown to be important for game performance in competitive male and female players (Mohr et al., 2016; Mohr et al., 2022). In these studies, in line with the findings above, NAK subunits and NHE1 were positively associated with high‐intensity game performance variables and with associations with different game performance indices also found for MCT4, HAD, CS, and myosin heavy chain composition (Mohr et al., 2016; Mohr et al., 2022). However, as ice hockey with the intense intermittent burst‐type efforts differs markedly from soccer or the 1–4 min all‐out efforts previously assessed, the role of various skeletal muscle phenotype variables for ice hockey specific performance is unknown.

The purpose of the present study was to test the main hypothesis that expression of skeletal muscle proteins involved in Na^+^ and K^+^ ion regulation (NAK subunits) and proton handling (NHE1) were positively associated with markers of game performance in elite male ice hockey players. In addition, it was hypothesized that the expression of type IIa myosin would be positively related to in‐game sprint performance. Secondarily, other markers of training status and muscle phenotype characteristics were included as more explorative assessments of potential in‐game performance indicators, while potential position‐specific differences were addressed.

## 2 MATERIALS AND METHODS

### 2.1 Participants

Sixteen male Danish U20 national team ice hockey players (Mean ± SD; age 19.4 ± 0.5 years; height 184 ± 6 cm; weight 85.2 ± 8.2 kg; body fat 14.4 ± 3.5%; muscle mass 41.8 ± 4.7 kg; Yo‐Yo Intermittent Recovery 1 Ice Hockey Test, Yo‐Yo IR1‐IH, 2329 ± 280 m) were recruited for the study and participated in an ice hockey training game. The study population consisted of six defensemen and 10 forwards. Participants were informed of potential risks, discomforts, and benefits associated with the study, before giving their written consent to participate. The study adhered to the Declaration of Helsinki and was approved by the Regional Committees on Health Research Ethics for Southern Denmark (Project‐ID: S‐20210063).

### 2.2 Study design

The study is a part of a larger project where muscle fatigue and recovery after an elite ice hockey game was investigated (Thorsteinsson et al., 2023). The players participated in an ice hockey training game consisting of three regulation length periods separated by 18 min of rest. Players refrained from high‐intensity physical activity 48 h prior to the game and were not allowed to consume caffeine 12 h prior to the game. In addition, players were not allowed to consume carbohydrates during the game but could drink water ad libitum. To induce a competitive milieu during the game, official referees officiated the game and spectators were allowed to attend. In addition, the game was played in the middle of the Danish regular season and few weeks prior to the final roster selection for the U20 World Championships 2021 in Denmark. Therefore, the players were deemed to be physically well prepared, which also was indicated by their Yo‐Yo IR1‐IH test scores compared to reference values (Vigh‐Larsen, Haverinen, Panduro, et al., 2020; Vigh‐Larsen et al., 2019) and highly motivated to perform at their best abilities. Game activity was measured continuously using a local positioning system and players wore a heart rate monitor with accelerometer. Three hours prior to the game, a muscle biopsy (70–120 mg wet weight) was obtained from m. vastus lateralis.

### 2.3 Monitoring of game activity and loading

The game load was continuously monitored using a local positioning system (LPS tracking; KINEXON Precision Technologies, KINEXON ONE, version 1.0, Munich, Germany) as previously described (Thorsteinsson et al., 2023). In brief, antennas were placed around the rink and players were equipped with a vest containing a sensor. To limit the number of comparisons performed, a selected few tracking variables were extracted including total distance skating (>1.0 km/h), high‐intensity skating (>17.0 km/h), number of accelerations and decelerations combined, and number of intense (>3.0 m/s^2^) accelerations and decelerations combined. Data were sampled at 20 Hz and further analyzed using KINEXON software. During the game, match activity recordings were not captured for three players during one period. Therefore, game activity relative to time on‐ice is based on two periods for these players. Heart rate was monitored during the game using a heart rate monitor with accelerometer (Team Polar, Polar Electro Oy, Kempele, Finland).

### 2.4 Performance measurements

1–2 weeks prior to the study the participants performed the Yo‐Yo IR1‐IH on the ice (Vigh‐Larsen et al., 2019). In brief, the test consisted of 2 × 20 m shuttles at increasing speeds where the players had to follow the tempo set by beeps from an audio recording. Subjects were given a warning the first time they were unable to reach the line in time, and the test was terminated on the second warning. Total distance covered was noted and represented the result. Due to logistical reasons and a busy game schedule three players were unable to participate in the Yo‐Yo IR1‐IH.

Prior to the game, players performed a repeated sprint test on the ice consisting of 5 × 33 m sprints (goal line to second blue line) separated with 25 s active rest where players skated back to the starting line as previously done in ice hockey (Vigh‐Larsen, Ermidis, Rago, et al., 2020). Players had the stick in one hand and were instructed to initiate the sprints 1 m before the starting line to avoid triggering the timing gates (Witty GateWireless Training Timer; Microgate, Italy) prematurely. Subjects were familiarized to the test on the day prior to the game by performing three repeated sprints of the test.

In addition, mechanical muscle neuromuscular function was assessed with maximal voluntary and electrically stimulated isometric contractions of the knee extensors m. quadriceps femoris using a special built chair (MID‐Chair) (Dynamometer, Science to Practice, (S2P), Ljubljana, Slovenia). The test procedure is described elsewhere (Thorsteinsson et al., 2023). Participants were familiarized to the test prior to the testing day and warmed up on a bicycle ergometer (Monark Ergomedic 874E, Monark, Sweden) at 100 W for 5 min prior to the test followed by specific warm up by knee‐extensor contractions seated in the chair.

### 2.5 Muscle analysis

The muscle tissue (70–120 mg w.w.) was immediately frozen in liquid nitrogen and subsequently stored at −80°C until further analysis. The muscle samples were weighed before and after freeze drying, and 1 h after freeze drying to correct for water uptake. The samples were dissected free of visible blood, connective tissue, and fat. Maximal citrate synthase (CS), phosphofructokinase (PFK), and beta‐hydroxyacyl‐CoA‐dehydrogenase (HAD) activities were determined fluorometrically as previously described (Seidemann et al., 1973). Each muscle sample was analyzed in triplicates.

### 2.6 Western blotting

Each dissected sample was divided into two tubes and homogenized in a fresh batch of cold homogenization buffer (10% glycerol, 20 Na‐pyrophosphate, 150 NaCl, 50 HEPES (pH 7.5), 1% NP‐40, 20 β‐glycerophosphate, 2 Na3VO4, 10 NaF, 2 PMSF, 1 EDTA (pH 8), 1 EGTA (pH 8), 10 μg/mL aprotinin, 10 μg/mL leupeptin, and 3 μg/mL benzamidine). Samples were homogenized in a tissuelyser (Qiagen Tissuelyser II, Retsch GmbH, Haan, Germany) 2 × 2 min at 28.5 Hz. Afterward, samples were rotated end over end for 1 h at 4°C, and thereafter sonicated (Branson Digital Sonifier) 1 × 10 s at 10% amplitude. The samples were then centrifuged at 18320 ***g*** for 20 min at 4°C, and the supernatant was carefully pipetted and used for further analysis. Total protein content in lysates was determined in triplicates with a standard BCA protein assay kit (71285, Millipore). The lysates were diluted to appropriate protein concentrations in a 6 × sample buffer (0.5 M Tris‐base, DTT, SDS, glycerol, and bromphenol blue) and MilliQ water (Milli‐Q® Reference system, Millipore). The protein expression was determined as previously described (Thomassen et al., 1985). In brief, an equal amount of total protein was loaded for each sample in different wells on precasted gels (TGX Stain‐Free™ Precast Gels, 5678035 and 5678085, Bio‐Rad Laboratories). Samples from the same subject were always loaded on the same gel. Gels were run for 1 h at 150 V and constant 0.06 A/gel where proteins were separated by SDS page gel electrophoresis. Each gel was semidry transferred to a PVDF membrane (Immobilon®‐P, IPVH00010, Millipore, Denmark). The membranes were then blocked in either 3% bovine serum albumin or 2% skimmed milk in Tris‐Buffered saline including 0.1% Tween‐20 (TBST) for 1 h. Thereafter, membranes were incubated in primary antibody at 4°C overnight. The membranes were then washed 2 × 10 min in TBST, and then incubated in secondary antibody for 1 h at room temperature. The membranes were washed for 3 × 15 min in TBST. The bands were visualized with ECL (Immobilon® Forte Western HRP substrate, WBLUF0500, Millipore), and band intensity was recorded with a digital camera (ChemiDoc MP Imaging System, Bio‐Rad Laboratories, USA). The intensity of the bands was quantified using Image Lab version 6.0 (Bio‐Rad Laboratories, USA), as previously described (Mohr et al., 2022). See Appendix S1 for membranes with molecular weight markers.

### 2.7 Antibodies

Prior to determining total protein content, the primary antibodies used in the study were optimized as previously described (Mohr et al., 2022). To determine protein content the following primary antibodies were used: Sodium‐potassium pump β_1_ isoform (NAKβ_1_): 40–45 kDa (MA3‐930, Affinity Bioreagents); Monocarboxylate transporter 4 (MCT4): 50 kDa (AB3316P, Millipore/Chemicon); Sodium‐hydrogen exchanger 1 (NHE1): 110 kDa (MAB3140, Chemicon); Myosin heavy chain isoform IIa (MHCIIa): 200 kDa (A4.74, Developmental Studies Hybridoma Bank, Iowa, USA); Chloride channel‐1 (ClC‐1): 110 kDa (ab189857, Abcam); Sodium‐potassium pump α_1_ isoform (NAKα_1_): 100 kDa (α6F Developmental Studies Hybridoma Bank, Iowa, USA); sodium‐potassium pump α_2_ isoform (NAK α_2_): 100 kDa (07–674 Millipore); ATP‐sensitive potassium channel (K_ATP_): 50 kDa (sc‐11,228, Santa Cruz Biotechnology, USA).

**FIGURE 1:**
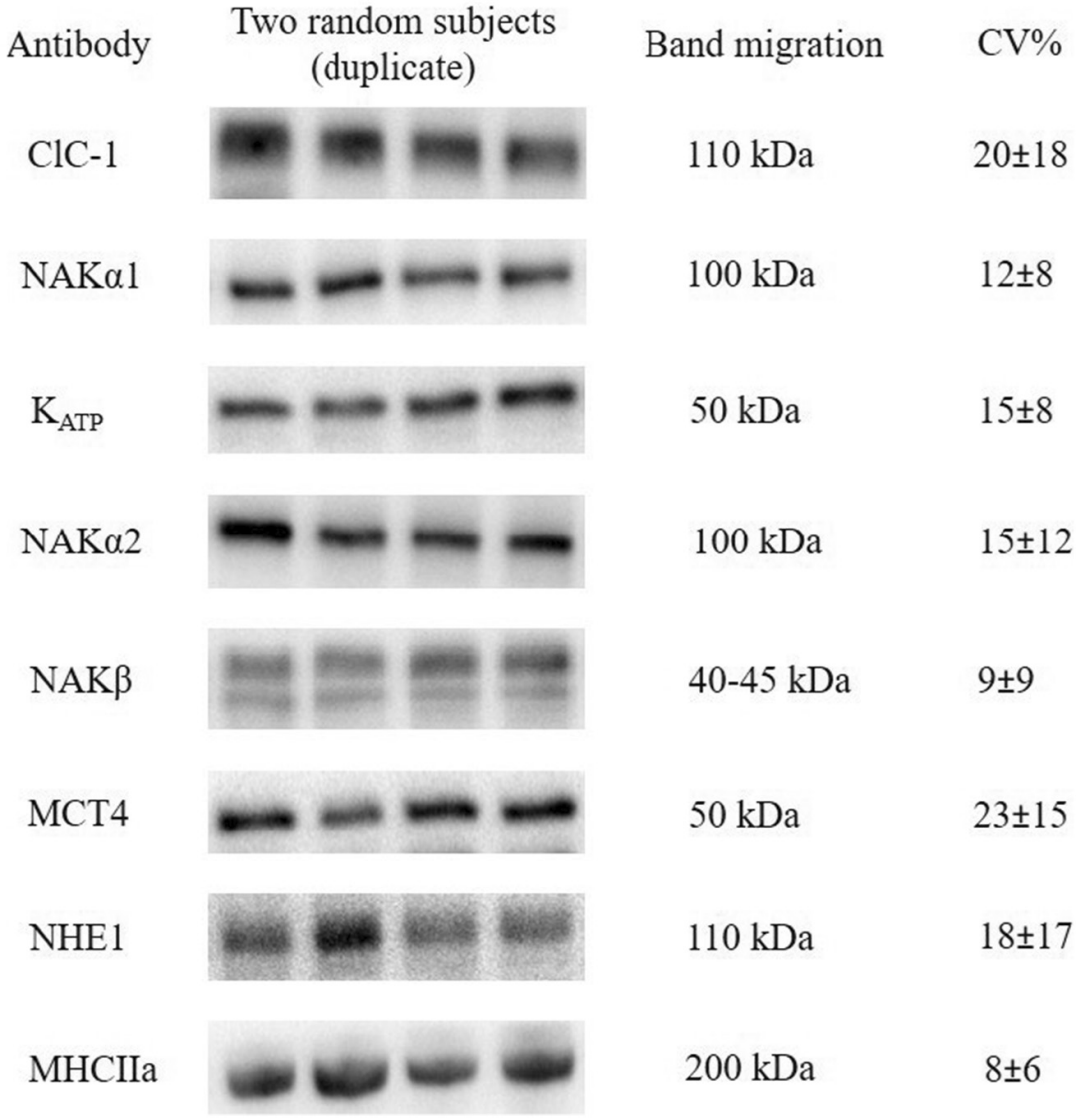
Representative image of total protein expression for the proteins measured in the present study. This figure shows images from two random subjects (duplicates) in the order “AABB”, molecular weight of band, and within subject's coefficient of variation (mean ± SD) of all samples from all participants.

The secondary antibodies used were HRP conjugated goat anti‐mouse, rabbit anti‐goat (P‐0447 and P‐0449 DAKO, Denmark), and goat anti‐rabbit (4010‐05 Southern Biotech, Birmingham AL, USA). Figure 1 shows a representative image of total protein expression for the proteins measured in the present study.

### 2.8 Statistics

The relationships between different selected variables were determined with Pearson's product–moment correlation coefficients. Correlations were run for the whole sample as well as for defensemen and forwards in isolation for each variable due to the position‐specific activity patterns. Correlations were considered as trivial (*r* = 0.1–0.29), moderate (*r* = 0.3–0.49), large (*r* = 0.5–0.69), very large (*r* = 0.7–0.89), nearly perfect (*r* = 0.9–0.99), and perfect (*r* = 1.0) in accordance with Hopkins et al. (2009). A significance level of *p* ≤ 0.05 was selected and all analysis were performed using Stata/IC16 (StataCorp, College Station, TX, USA) and graphs created with GraphPad Prism 9 (GraphPad Software, La Jolla, CA, USA). Significant correlations and correlations approaching statistical significance (*p* ≤ 0.10) are depicted with a regression line. Data are presented as means ± SD unless otherwise stated.

## 3 RESULTS

### 3.1 Game response

Average game data are presented in Table 1. Of note, positional differences were present with forwards covering less total distance than defensemen (4214 ± 511 m vs. 4887 ± 536 m, *p* = 0.058) but more high‐intensity skating (2360 ± 318 m vs. 1639 ± 341 m, *p* = 0.004). When expressed relative to time on‐ice this yielded a higher total distance covered per min in forwards compared to defensemen (224 ± 11 m/min vs. 178 ± 9 m/min, *p* < 0.001), as well as higher high‐intensity distance covered per min (126 ± 8 m/min vs. 62 ± 9 m/min, *p* < 0.001).

**TABLE 1:** Descriptive game activity profile presented as means ± SD (*n* = 16, unless otherwise stated).

Category	Total	Forwards	Defensemen	*p* Value
On‐ice time (min)	21.5 ± 5 (*n* = 13)	18.9 ± 2.8 (*n* = 9)	27.3 ± 2.0 (*n* = 4)	<0.001
Total distance (>1.0 km/h) (m)	4421 ± 601 (*n* = 13)	4214 ± 536 (*n* = 9)	4887 ± 511 (*n* = 4)	0.058
High‐intensity distance (>17.0 km/h) (m)	2138 ± 465 (*n* = 13)	2360 ± 318 (*n* = 9)	1639 ± 341 (*n* = 4)	0.004
Total acc + dec (*n*)	87 ± 18 (*n* = 13)	80 ± 16 (*n* = 9)	102 ± 12.3 (*n* = 4)	0.047
Intense acc + dec (>3.0 m/s^2^) (*n*)	40 ± 10 (*n* = 13)	38 ± 11 (*n* = 9)	43 ± 12 (*n* = 4)	0.415
Distance per min (m/min)	207 ± 25	224 ± 11 (*n* = 10)	178 ± 9 (*n* = 6)	<0.001
High‐intensity distance per min (m/min)	102 ± 33	126 ± 8 (*n* = 10)	62 ± 9 (*n* = 6)	<0.001
Acc + dec per min (*n*/min)	4.0 ± 0.9	4.0 ± 1.0 (*n* = 10)	3.9 ± 0.6 (*n* = 6)	0.839
Intense acc + dec per min (>3.0 m/s^2^) (*n*/min)	1.8 ± 0.6	1.9 ± 0.7 (*n* = 10)	1.6 ± 0.4 (*n* = 6)	0.362

### 3.2 Positional muscle phenotype differences

No significant differences were observed in muscle phenotype measures between positional roles (see Table 2, *p* = 0.100–0.991), despite forwards in general exhibiting higher absolute protein expression than defensemen. Moreover, no differences were present in Yo‐Yo IR1‐IH performance in forwards versus defensemen (2337 ± 326 vs. 2310 ± 174, *p* = 0.877), mean repeated sprint ability (4.71 ± 0.13 vs. 4.78 ± 0.16, *p* = 0.391) or peak sprint ability (4.59 ± 0.13 vs. 4.68 ± 0.21, *p* = 0.300).

**TABLE 2:** Playing position and skeletal muscle phenotype presented as means ± SD.

Protein/enzyme activity	Forwards	Defensemen	*p* Value
NAKα_1_	0.96 ± 0.47	0.61 ± 0.27	0.123
NAKα_2_	0.83 ± 0.15	0.66 ± 0.25	0.100
NAKβ_1_	0.78 ± 0.16	0.80 ± 0.21	0.917
NAK∑	2.58 ± 0.60	2.07 ± 0.63	0.129
NHE1	0.87 ± 0.23	0.72 ± 0.15	0.180
MCT4	0.97 ± 0.37	0.62 ± 0.52	0.138
MHCIIa	0.97 ± 0.38	0.78 ± 0.19	0.286
K_ATP_	0.97 ± 0.25	0.74 ± 0.31	0.136
ClC‐1	1.00 ± 0.31	0.76 ± 0.18	0.104
CS enzyme activity	41.1 ± 8.1	41.1 ± 8.1	0.991
HAD enzyme activity	24.0 ± 3.5	25.7 ± 3.6	0.410
PFK enzyme activity	490.8 ± 27.0	459.8 ± 65.5	0.238

### 3.3 Muscle phenotype and total in‐game skating performance

**FIGURE 2:**
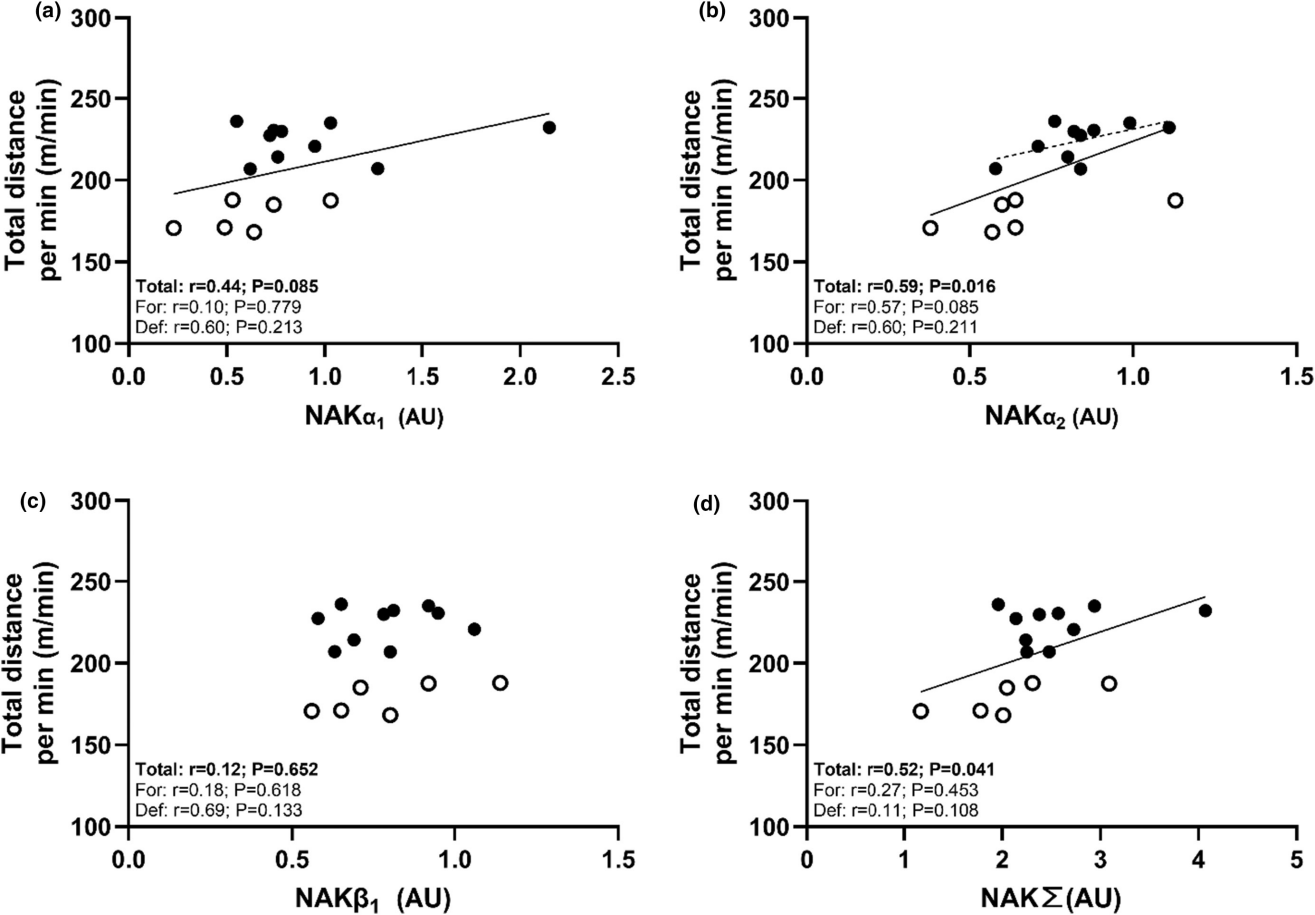
Individual values for distance covered per minute by players plotted against vastus lateralis NAK subunits (a, b, and c), as well as the sum of NAK isoforms (d). Plots show individual values and best fitted lines with a solid line depicting the whole sample and dotted lines positions‐specific regressions. Open symbols show individual values for defensemen and closed symbols show individual values for forwards.

**FIGURE 3:**
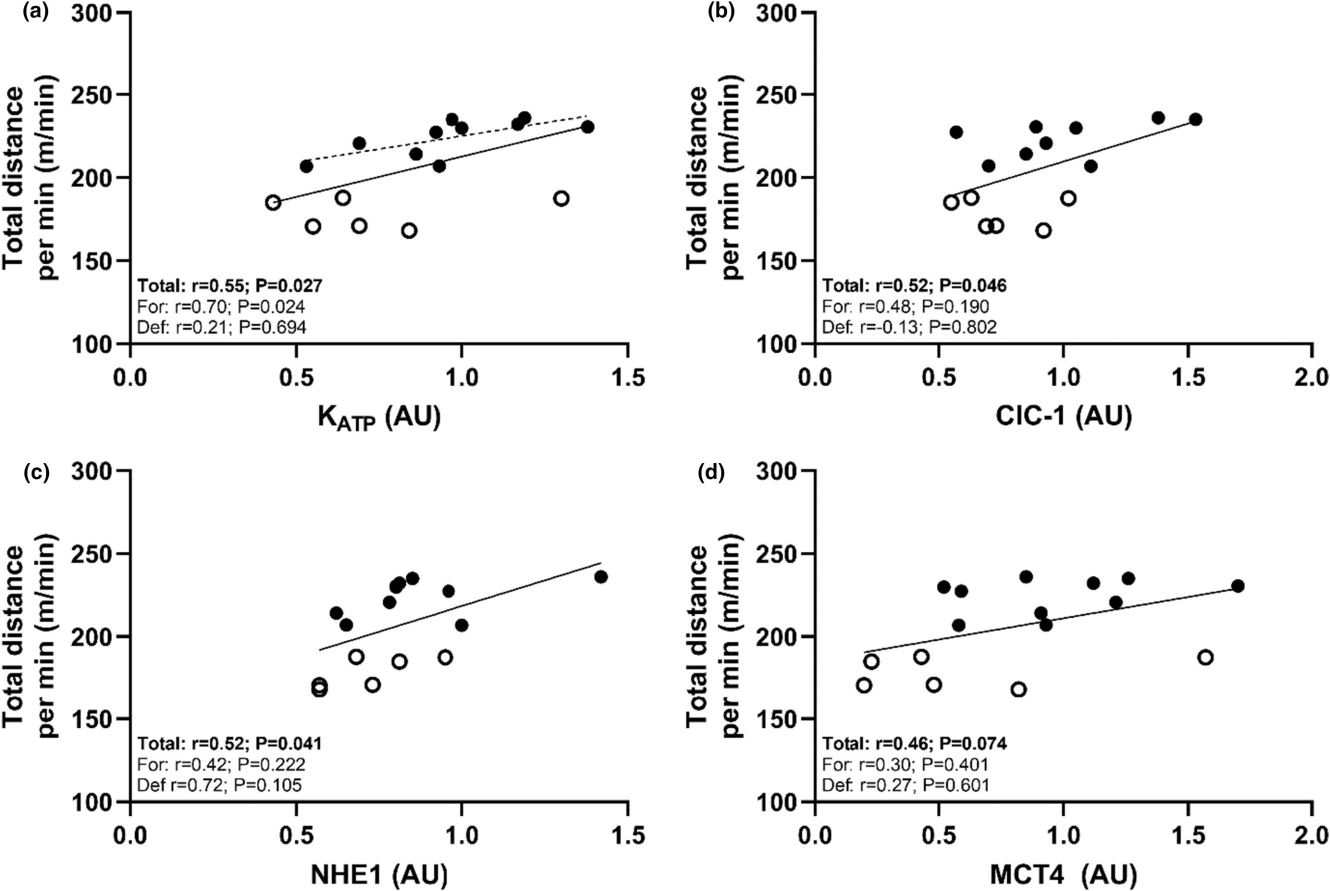
Individual values for distance covered per minute by players plotted against vastus lateralis proteins (a) K_ATP_, (b) CLC‐1, (c) NHE1, and (d) MCT4. Plots show individual values and best fitted lines with a solid line depicting the whole sample and dotted lines positions‐specific regressions. Open symbols show individual values for defensemen and closed symbols show individual values for forwards.

The sum of NAK subunits (NAK∑) was strongly associated with skating distance per min (see Table 3 and Figure 2). This was mediated primarily by a strong association between NAKα_2_ and skating distance per min and a trend (*p* = 0.085) toward a moderate association for NAKα_1_. On the contrary, NAKβ_1_ was unrelated to skating distance (*p* = 0.651). In addition, K_ATP_, NHE1, and ClC‐1 protein expression was strongly related to skating distance per min, while MCT4 exhibited a trend (*p* = 0.074) toward a moderate relationship with skating distance (Figure 3). However, when assessed within each positional role, these relationships only persisted for NAKα_2_ and K_ATP_ within forwards. No aerobic enzyme activities were related to distance covered per min across groups, however, within forwards a strong significant relationship was present for CS. In addition, Yo‐Yo‐IR1‐IH distance, mean repeated sprint ability and MVIC performance did not associate with total distance covered per min (*r* = 0.35; *p* = 0.236, *r* = −0.32; *p* = 0.231 and *r* = −0.15; *p* = 0.582) at the group‐level. However, a strong positive association was present for Yo‐Yo‐IR1‐IH distance and total distance covered per min for forwards (*r* = 0.89; *p* = 0.001) and with Yo‐Yo‐IR1‐IH distance significantly associated with CS enzyme activity (*r* = 0.71; *p* = 0.009).

**TABLE 3:** Correlations of muscle proteins or enzyme activities and total distance per min.

Muscle protein/enzyme variable	Correlation coefficient, *p* value and participant numbers
NAKα_1_	Total *r* = 0.44; *p* = 0.085 (*n* = 16) Forwards *r* = 0.10; *p* = 0.779 (*n* = 10) Defensemen *r* = 0.60; *p* = 0.213 (*n* = 6)
NAKα_2_	Total *r* = 0.59; *p* = 0.016 (*n* = 16) Forwards *r* = 0.59; *p* = 0.085 (*n* = 10) Defensemen *r* = 0.60; *p* = 0.211 (*n* = 6)
NAKβ_1_	Total *r* = 0.12; *p* = 0.652 (*n* = 16) Forwards *r* = 0.18; *p* = 0.618 (*n* = 10) Defensemen *r* = 0.69; *p* = 0.133 (*n* = 6)
NAK∑	Total *r* = 0.52; *p* = 0.041 (*n* = 16) Forwards *r* = 0.27; *p* = 0.453 (*n* = 10) Defensemen *r* = 0.11; *p* = 0.108 (*n* = 6)
NHE1	Total *r* = 0.52; *p* = 0.041 (*n* = 16) Forwards *r* = 0.42; *p* = 0.222 (*n* = 10) Defensemen *r* = 0.72; *p* = 0.105 (*n* = 6)
MCT4	Total *r* = 0.46; *p* = 0.074 (*n* = 16) Forwards *r* = 0.30; *p* = 0.401 (*n* = 10) Defensemen *r* = 0.26; *p* = 0.601 (*n* = 6)
MHCIIa	Total *r* = 0.34; *p* = 0.195 (*n* = 16) Forwards *r* = 0.12; *p* = 0.735 (*n* = 10) Defensemen *r* = 0.61; *p* = 0.202 (*n* = 6)
K_ATP_	Total *r* = 0.55; *p* = 0.027 (*n* = 16) Forwards *r* = 0.70; *p* = 0.024 (*n* = 10) Defensemen *r* = 0.21; *p* = 0.695 (*n* = 6)
ClC‐1	Total *r* = 0.52; *p* = 0.046 (*n* = 15) Forwards *r* = 0.48; *p* = 0.190 (*n* = 9) Defensemen *r* = −0.13; *p* = 0.802 (*n* = 6)
CS enzyme activity	Total *r* = 0.16; *p* = 0.573 (*n* = 14) Forwards *r* = 0.75; *p* = 0.019 (*n* = 9) Defensemen *r* = −0.25; *p* = 0.679 (*n* = 5)
HAD enzyme activity	Total *r* = −0.19; *p* = 0.523 (*n* = 14) Forwards *r* = 0.30; *p* = 0.431 (*n* = 9) Defensemen *r* = −0.32; *p* = 0.600 (*n* = 5)
PFK enzyme activity	Total *r* = 0.29; *p* = 0.335 (*n* = 13) Forwards *r* = 0.18; *p* = 0.674 (*n* = 8) Defensemen *r* = −0.34; *p* = 0.57 (*n* = 5)

### 3.4 Muscle phenotype and high‐intensity skating performance

**FIGURE 4:**
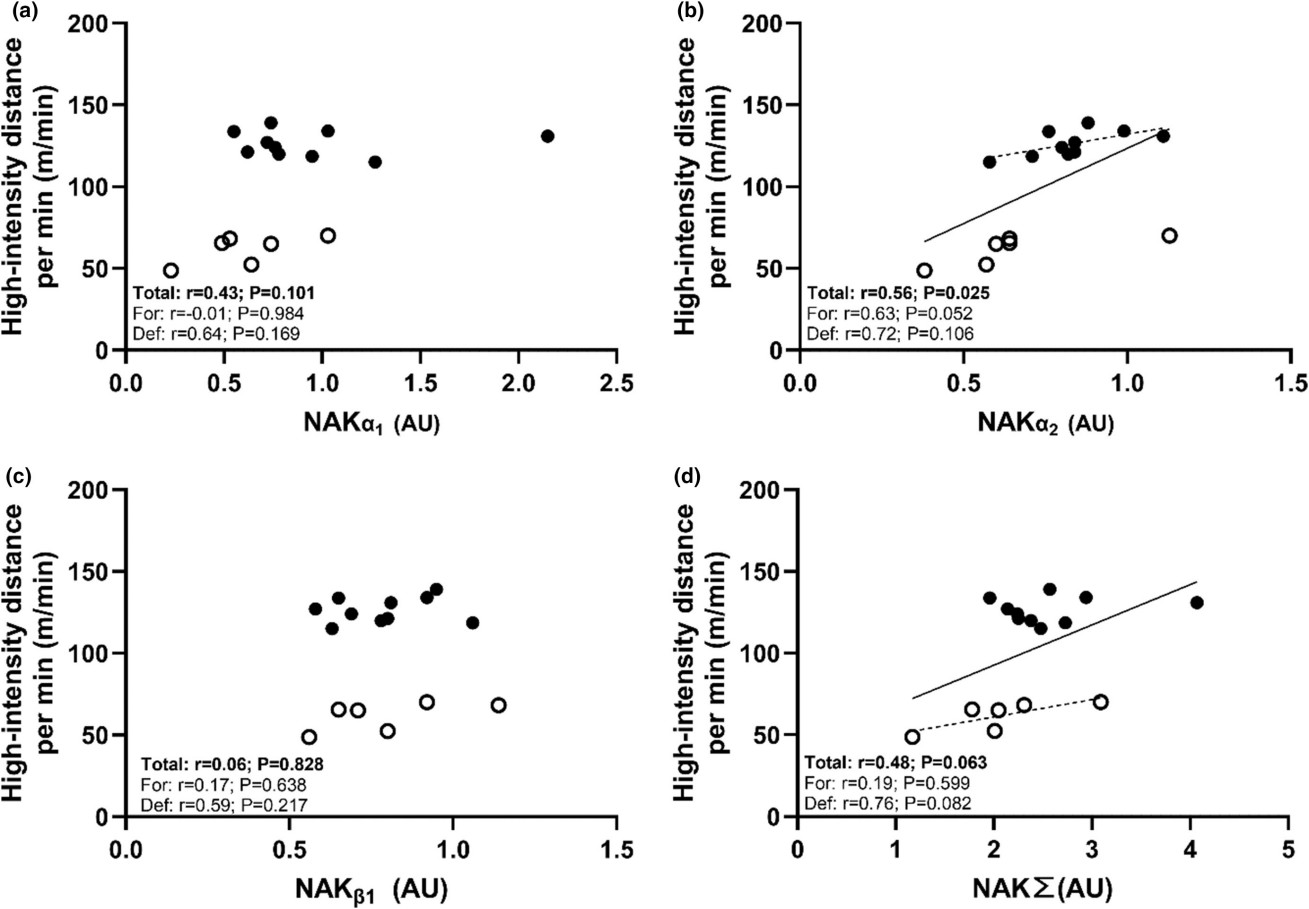
Individual values for high‐intensity (>17 km/h) distance covered per minute by players plotted against vastus lateralis NAK subunits (a, b, and c), as well as the sum of NAK isoforms (d). Plots show individual values and best fitted lines with a solid line depicting the whole sample and dotted lines positions‐specific regressions. Open symbols show individual values for defensemen and closed symbols show individual values for forwards.

**FIGURE 5:**
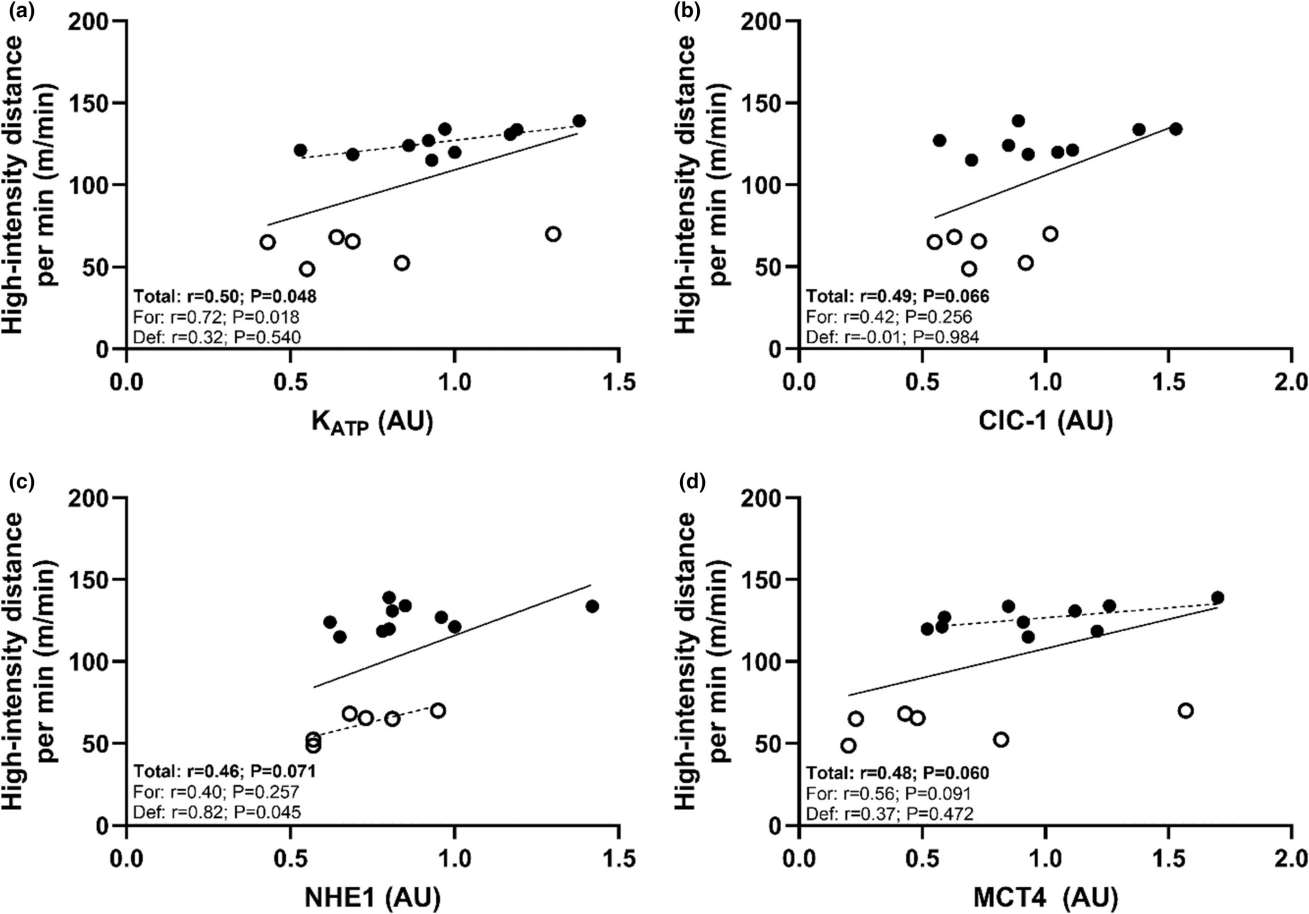
Individual values for distance covered per minute by players plotted against vastus lateralis proteins (a) K_ATP_, (b) CLC‐1, (c) NHE1, and (d) MCT4. Plots show individual values and best fitted lines with a solid line depicting the whole sample and dotted lines positions‐specific regressions. Open symbols show individual values for defensemen and closed symbols show individual values for forwards.

High‐intensity skating distance (>17.0 km/h) per min correlated strongly with the expression of NAKα_2_ (Table 4 and Figure 4) but not with any other NAK subunits. This relationship persisted when examined within the forward positional group. Totaling the sum of NAK subunits resulted in a moderate association, approaching statistical significance only (*p* = 0.063). Protein expression of NHE1, MCT4, K_ATP_ channels, and ClC‐1 all exhibited moderate‐to‐strong associations with high‐intensity skating per min with borderline statistical significance ranging from *p* = 0.048 to *p* = 0.071 (Figure 5). However, this association was only consistent when examined within forwards for K_ATP_. No relationship was found for MHCIIa expression and high‐intensity skating distance or peak speeds attained in‐game or during the repeated sprint test (*r* = 0.26; *p* = 0.342 and *r* = 0.26; *p* = 0.341, respectively). Similarly, Yo‐Yo IR1 performance and mean repeated sprint ability did not associate with high‐intensity skating distance (*r* = 0.14; *p* = 0.646 and *r* = −0.28; *p* = 0.291, respectively). This was also the case when evaluated within positions where only Yo‐Yo IR1 distance covered for forwards approached a positive relationship with high‐intensity distance per min (*r* = 0.61; *p* = 0.140). Finally, knee‐extensor rate of torque (RTD) obtained from the 20 and 50 Hz stimulations exhibited strong significant correlations with in‐game peak sprint performance (*r* = 0.62; *p* = 0.010 and *r* = 0.53; *p* = 0.033), as well as peak fastest time during the repeated sprint test (*r* = −0.57; *p* = 0.022 and *r* = −0.59; *p* = 0.015). However, this was not the case for RTD obtained during the MVIC (*r* = 0.17–0.33; *p* = 0.54–0.22).

**TABLE 4:** Correlations of muscle proteins or enzyme activities and high‐intensity skating distance per min.

Muscle protein/enzyme variable	Correlation coefficient, *p* value and participant numbers
NAKα_1_	Total *r* = 0.43; *p* = 0.101 (*n* = 16) Forwards *r* = −0.01; *p* = 0.984 (*n* = 10) Defensemen *r* = 0.64; *p* = 0.169 (*n* = 6)
NAKα_2_	Total *r* = 0.56; *p* = 0.025 (*n* = 16) Forwards *r* = 0.63; *p* = 0.052 (*n* = 10) Defensemen *r* = 0.72; *p* = 0.106 (*n* = 6)
NAKβ_1_	Total *r* = 0.06; *p* = 0.828 (*n* = 16) Forwards *r* = 0.17; *p* = 0.638 (*n* = 10) Defensemen *r* = 0.59; *p* = 0.217 (*n* = 6)
NAK∑	Total *r* = 0.48; *p* = 0.063 (*n* = 16) Forwards *r* = 0.19; *p* = 0.599 (*n* = 10) Defensemen *r* = 0.76; *p* = 0.082 (*n* = 6)
NHE1	Total *r* = 0.46; *p* = 0.071 (*n* = 16) Forwards *r* = 0.40; *p* = 0.257 (*n* = 10) Defensemen *r* = 0.82; *p* = 0.045 (*n* = 6)
MCT4	Total *r* = 0.48; *p* = 0.060 (*n* = 16) Forwards *r* = 0.56; *p* = 0.091 (*n* = 10) Defensemen *r* = 0.37; *p* = 0.472 (*n* = 6)
MHCIIa	Total *r* = 0.33; *p* = 0.208 (*n* = 16) Forwards *r* = 0.17; *p* = 0.639 (*n* = 10) Defensemen *r* = 0.57; *p* = 0.321
K_ATP_	Total *r* = 0.50; *p* = 0.048 (*n* = 16) Forwards *r* = 0.72; *p* = 0.018 (*n* = 10) Defensemen *r* = 0.32; *p* = 0.540 (*n* = 6)
ClC‐1	Total *r* = 0.49; *p* = 0.066 (*n* = 15) Forwards *r* = 0.42; *p* = 0.256 (*n* = 9) Defensemen *r* = −0.01; *p* = 0.984 (*n* = 6)
CS enzyme activity	Total *r* = 0.07; *p* = 0.818 (*n* = 14) Forwards *r* = 0.54; *p* = 0.133 (*n* = 9) Defensemen *r* = −0.18; *p* = 0.778 (*n* = 5)
HAD enzyme activity	Total *r* = −0.22; *p* = 0.445 (*n* = 14) Forwards *r* = 0.21; *p* = 0.584 (*n* = 9) Defensemen *r* = −0.25; *p* = 0.684 (*n* = 5)
PFK enzyme activity	Total *r* = 0.32; *p* = 0.292 (*n* = 13) Forwards *r* = −0.28; *p* = 0.496 (*n* = 8) Defensemen *r* = −0.05; *p* = 0.931 (*n* = 5)

### 3.5 Muscle proteins and accelerations and deceleration game profile

The association between muscle proteins and total acceleration and deceleration movement profile relative to time on‐ice exhibited a very similar pattern as that of intense acceleration and deceleration movements and only the former is therefore depicted (Table 5). None of the muscle proteins measured were significantly associated with the number of accelerations and decelerations per minute. Further, neither Yo‐Yo IR1‐IH performance (*r* = −0.17; *p* = 0.579) nor MVIC torque (*r* = −0.47; *p* = 0.065) were associated with the acceleration and deceleration density, although the latter approached statistical significance in the negative direction.

**TABLE 5:** Correlations of muscle proteins or enzyme activities and total number of accelerations and decelerations per min.

Muscle protein/enzyme variable	Correlation coefficient, *p* value and participant numbers
NAKα_1_	Total *r* = −0.21; *p* = 0.426 (*n* = 16) Forwards *r* = −0.34; *p* = 0.331 (*n* = 10) Defensemen *r* = 0.17; *p* = 0.744 (*n* = 6)
NAKα_2_	Total *r* = −0.15; *p* = 0.581 (*n* = 16) Forwards *r* = −0.30; *p* = 0.398 (*n* = 10) Defensemen *r* = −0.07; *p* = 0.892 (*n* = 6)
NAKβ_1_	Total *r* = −0.09; *p* = 0.740 (*n* = 16) Forwards *r* = −0.33; *p* = 0.347 (*n* = 10) Defensemen *r* = 0.42; *p* = 0.411 (*n* = 6)
NAK∑	Total *r* = −0.22; *p* = 0.422 (*n* = 16) Forwards *r* = −0.43; *p* = 0.216 (*n* = 10) Defensemen *r* = 0.18; *p* = 0.733 (*n* = 6)
NHE1	Total *r* = 0.27; *p* = 0.318 (*n* = 16) Forwards *r* = 0.31; *p* = 0.388 (*n* = 10) Defensemen *r* = 0.08; *p* = 0.885 (*n* = 6)
MCT4	Total *r* = −0.36; *p* = 0.171 (*n* = 16) Forwards *r* = −0.51; *p* = 0.130 (*n* = 10) Defensemen *r* = −0.30; *p* = 0.562 (*n* = 6)
MHCIIa	Total *r* = −0.18; *p* = 0.500 (*n* = 16) Forwards *r* = −0.25; *p* = 0.478 (*n* = 10) Defensemen *r* = 0.05; *p* = 0.922 (*n* = 6)
K_ATP_	Total *r* = −0.36; *p* = 0.170 (*n* = 16) Forwards *r* = −0.46; *p* = 0.186 (*n* = 10) Defensemen *r* = −0.40; *p* = 0.436 (*n* = 6)
CLC‐1	Total *r* = 0.28; *p* = 0.318; (*n* = 15) Forwards *r* = 0.41; *p* = 0.275 (*n* = 9) Defensemen *r* = −0.49; *p* = 0.326 (*n* = 6)
CS	Total *r* = −0.07; *p* = 0.801 (*n* = 14) Forwards *r* = 0.09; *p* = 0.813 (*n* = 9) Defensemen *r* = −0.83; *p* = 0.084 (*n* = 5)
HAD	Total *r* = −0.24; *p* = 0.403 (*n* = 14) Forwards *r* = −0.23; *p* = 0.56 (*n* = 9) Defensemen *r* = −0.55; *p* = 0.337 (*n* = 5)
PFK	Total *r* = −0.238; *p* = 0.430 (*n* = 13) Forwards *r* = 0.05; *p* = 0.908 (*n* = 8) Defensemen *r* = −0.94; *p* = 0.018 (*n* = 5)

## 4 DISCUSSION

In this first study exploring the associations between skeletal muscle phenotypes and on‐ice skating performance in elite male ice hockey players, we demonstrate relations between some key proteins involved in skeletal muscle ion and metabolic regulation and on‐ice performance. Thus, the most persisting correlations, also evident within a distinct positional role, were present for NAKα_2_ and K_ATP_, which were associated with both total and high‐intensity distance metrics. Moreover, strong significant associations were found within forwards for CS enzyme activity and total distance covered, as well as Yo‐Yo IR1‐IH distance and total distance covered, supporting an important role of aerobic capacity for intermittent exercise performance. However, contrary to our hypothesis, less clear associations were found for the remaining Na^+^‐K^+^‐ATPase subunits and lactate and H^+^ exchangers, possibly indicating that the hybrid nature and/or variability of ice hockey game performance and physiological capacity does not clearly favor distinct muscle phenotype traits. In line with this, no association was found for type IIa myosin heavy chain expression and in‐game sprint performance in contrast to our hypothesis.

### 4.1 Associations to ice hockey endurance performance

The muscle protein expression of NAK∑, NAKα_2_, K_ATP_, NHE1, and ClC‐1 were all strongly associated with distance covered per min suggesting that the regulation of K^+^ and H^+^ muscle homeostasis may be important for endurance capacity during an ice hockey game. However, this relationship did only persist for NAKα_2_ and K_ATP_ in forwards when evaluated within distinct positions. These findings contrast those in male soccer players where protein expression of HAD displayed the strongest correlation with total game distance (Mohr et al., 2016), whereas the present study demonstrated no correlation between HAD enzyme activity and total distance covered per minute. This would suggest that muscle fat oxidation is a less important factor for endurance capacity in ice hockey, in line with the high‐intense activity pattern and great reliance on endogenous glycogen stores (Lignell et al., 2018; Vigh‐Larsen et al., 2021; Vigh‐Larsen, Ermidis, Rago, et al., 2020). Moreover, CS maximal activity, as well as Yo‐Yo IR1‐IH distance were strongly related to total distance covered within forwards suggesting an important role of aerobic metabolism for intermittent exercise performance in line with the literature (Glaister, 2005; Vigh‐Larsen & Mohr, 2024). Indeed, aerobic capacity may be important both to limit the taxation of anaerobic energy systems and to facilitate recovery (e.g., phosphocreatine resynthesis) between efforts (Balsom et al., 1994; Sahlin et al., 1979).

### 4.2 Associations to ice hockey high‐intensity performance

The proteins most closely related to distance covered per minute at fast skating speeds (>17.0 km/h) were NAKα_2_ and K_ATP_ protein expression which both exhibited strong associations, also when examined within positional roles (for forwards). In addition, moderate and borderline significant overall associations appeared for NAK∑, NHE1, MCT4, and ClC‐1 inducing a comparable pattern to that of total skating distance, suggesting that the muscle phenotype characteristics underpinning total and high‐intensity skating performance in ice hockey are comparable entities. Importantly, a nearly perfect correlation was present between total distance per minute and total high‐intensity skating per minute (*r* = 0.96; *p* < 0.001) clearly showing that these two factors are highly interrelated in ice hockey (with the percentage of total distance covered at high skating speeds ranging from 29% to 57% of the total distance at the individual level).

The association between NAK subunits and high‐intensity skating is in accordance with findings in male soccer players (Mohr et al., 2016) where skeletal muscle abundance of these proteins explained 50% of the variance in high‐speed running during the most intense periods of a game, which may resemble the demands commonly endured during ice hockey shifts. Also, Iaia et al. (2011) found the NAKβ_1_ subunit to explain 11%–34% of the variance in performance during exercise periods lasting 45 s to 4 min. Finally, a myriad of training studies demonstrate parallel upregulation of high‐intensity intermittent exercise performance and NAK protein expression (for review see (Hostrup et al., 2021; Hostrup & Bangsbo, 2017)). Importantly, some discrepancy exists since in the present study the NAKβ_1_ subunit exhibited no correlations with either total distance or high‐intensity distance covered. This is in clear opposition to the results obtained by Iaia et al. (2011) where it was the abundance of the NAKβ_1_ subunit, in particular, which was associated with intense exercise performance. Furthermore, in the study by Mohr et al. (2016), both NAKα_2_ and NAKβ_1_ were strongly associated with peak 5‐min high‐intensity performance, whereas no correlation was reported for NAKα_1_. Similarly, in the study by Mohr et al. (2022), only NAKβ_1_ was reported to associate with high‐intensity running performance. Thus, no clear pattern seems to be apparent for the role and importance of each NAK subunit for intense exercise performance and the causality of these relationships therefore requires further investigation. In this regard, it is important to consider that the Western blot methodology employed here does not allow direct quantification of functional NAK units which consist of both alpha‐, beta‐, and regulatory FXYD1‐subunits (Thomassen et al., 1985). However, studies quantifying functional NAK using ouabain‐binding also point to a correlation between NAK concentration in muscle and sprint performance (McKenna et al., 1993). Of note, specifically the α_2_ subunit which was associated with performance in the present study has previously been suggested to represent the major pool of Na^+^‐K^+^‐ATPase of skeletal muscle essential for contractility and fatigue resistance, conceivably associated with a primary loci in the transverse tubules (Radzyukevich et al., 2013).

K^+^ efflux from the myocytes during intense exercise does also occur via K_ATP_ channels, with the opening probability of these channels increasing with a reduction in intramuscular ATP and pH (Davies, 1990). Muscle ATP is likely to decrease transiently during the high intensity periods of an ice hockey game as has been observed during lab‐based exercise of a similar type (e.g., single 6‐s sprint) (Gaitanos et al., 1993), and muscle pH has been shown to decrease moderately after shifts, which will be sensed by these channels leading to continuous modulations of their opening state (Vigh‐Larsen, Ermidis, Rago, et al., 2020). In our study, we saw significant correlations (*r* = 0.50–0.55) between high‐speed skating per min and total distance per min and the protein expression of the Kir6.2 K_ATP_ channel, which were even stronger within the forward positional role (*r* = 0.70–0.72), as well as a relationship of a similar magnitude between Yo‐Yo‐IR1‐IH test performance and the Kir6.2 channel (*r* = 0.59, *p* = 0.034). Notably, a higher abundance of these channels may theoretically increase K^+^ efflux during intense exercise when ATP and pH can be lowered, which would contribute to a loss of excitability, contrasting the direction of our findings. It may, however, be speculated that the K_ATP_ channels are being upregulated in parallel to the NAK ATPase, as a potential protective mechanism to counter fatigue‐induced cell damage (Hostrup et al., 2021). However, in healthy young males no change was observed in K_ATP_ abundance despite a 15% increase in the NAKα_2_ subunit following high‐intensity training (Nielsen et al., 2004), whereas in fact a 14% decrease was observed after a 7‐week intensified training period in trained cyclists with no change in NAKα_2_ (Gunnarsson et al., 2013). Accordingly, the role of the absolute abundance of K_ATP_ channel proteins in performance and ion homeostasis is not yet clear.

In line with this, a strong positive correlation was observed between the protein expression of the muscle specific chloride channel, ClC‐1, and total skating distance covered per min and a tendency toward a relationship for high‐intensity skating distance (*p* = 0.066). This is in contrast with a study of elite women soccer players, where an inverse correlation was shown between ClC‐1 and high‐speed running at the end of a game (Mohr et al., 2022). However, reductions in Cl^−^ conductance through decreases in ClC‐1 opening state during contractile activity in vitro counteracts the negative effects of interstitial K^+^ accumulation, while prolonged fatiguing activity leads to a sudden opening of ClC‐1 channels and a loss of muscle excitability (Baekgaard Nielsen et al., 2017; Leermakers et al., 2020; Pedersen et al., 2009; Pedersen et al., 2016). It is therefore not straightforward that an increased abundance of ClC‐1 channels would associate to improved performance. Indeed, a previous study reported a negative correlation between ClC‐1 abundance and peak power output during an incremental test to exhaustion (VO_2max_ test) (Thomassen et al., 2018). These results contrasts those of the present study where overall correlations between ClC‐1 abundance and total and high‐intensity skating were present, as well as a positive (*r* = 0.42) but nonsignificant (*p* = 0.173) relationship with incremental test performance during the Yo‐Yo IR1‐IH test. However, these correlations did not persist within distinct positions indicating that these could represent spurious findings. Similarly, no association was previously found between ClC‐1 abundance and knee‐extensor strength endurance capacity during repetitions to failure at 30% of 1RM in healthy, untrained individuals underscoring the ambiguity of the role of ClC‐1 abundance in fatigue resistance (Wang et al., 2023).

In the present study, the protein expression of MCT4 tended to correlate moderately with total distance covered per minute (*p* = 0.074) and high‐intensity distance covered per minute (*p* = 0.060), while the protein expression of NHE1 correlated strongly with total skating distance and tended (*p* = 0.076) to correlate moderately with performance at higher intensities. It has previously been shown that there are substantial increases in muscle lactate during an ice hockey game, while muscle pH is moderately reduced at the whole‐muscle level (with more severe reductions possible in sub‐groups of fibers) (Vigh‐Larsen, Ermidis, Rago, et al., 2020). Although still debated, such metabolic perturbations could be implicated in performance‐deteriorations (Fitts, 1994; Nielsen et al., 2001; Overgaard et al., 2010). However, relationships were not consistently maintained at a significant level when evaluated within distinct positional roles, although the direction of the relationships were unaltered. It is therefore unclear whether these relationships are important or not. In soccer, MCT subunits and NHE1 have been shown to correlate with game performance measures (Mohr et al., 2016; Mohr et al., 2022), with NHE1 being the strongest predictor of high‐intensity intermittent running performance in an elite women's soccer game (Mohr et al., 2022). In accordance, NHE1 has been shown to explain 24% of the variance in performance during all‐out exercise lasting 45 s (Iaia et al., 2011), which corresponds to a shift in ice hockey. However, in contrast, no associations were present for exercise for 30 s or 60 s duration, highlighting some heterogeneity in the results. Taken together, further studies directly assessing the capacity to perform ice hockey‐specific intense sequences in association with muscle phenotype measures would be important to obtain more clear results and devoid the potential influence of technical/tactical aspects.

### 4.3 Muscle fiber type, lower body strength, and sprint activities

In contrast to our hypothesis, no relationship was present between myosin heavy chain type IIa expression and high‐intensity exercise performance metrics including high‐intensity skating distance per minute or peak speeds attained during the repeated sprint test or during the game. In comparison Iaia et al. (2011) found that the proportion of type IIx fibers was associated with 30‐s intense exercise performance, whereas fiber type composition was reported to be the muscle parameter of greatest importance for sprint performance during a soccer match in males (Mohr et al., 2016), as well as in female players (Mohr et al., 2022). In the present study, myosin heavy chain composition was assessed from a single muscle biopsy and it may be that the variability in fiber type across sampling sites may not have enabled us with the precision to detect possible associations (Horwath et al., 2021). Also, we only measured the type IIa isoform, which is a limitation. Finally, lean leg muscle mass may be a stronger predictor of sprint performance as suggested by previous investigations during cycling sprints (Galvan‐Alvarez et al., 2024; Kordi et al., 2020; van der Zwaard et al., 2018). We were not able to obtain a measure of lean leg muscle mass in the present study; however, knee‐extensor MVIC was unrelated to all distance variables, including sprinting distance and peak sprint performance in‐game or during the repeated sprint test.

Collectively, our correlation analyses revealed some interesting relationships that may explain part of the variance in elite ice hockey game performance in support of an importance of the ability to maintain ionic and metabolic perturbations within a range where they do not negatively influence intense exercise performance. However, several of the proteins of interest exhibited no clear association with performance, opposing a clear role of distinct muscle phenotype traits to excel in a hybrid sport like ice hockey. Moreover, it should be acknowledged that the present results are based on associations, and it will be interesting to conduct future studies to support these findings with more direct assessments of maximal intermittent ice hockey‐specific exercise capacity of varying duration in association with muscle phenotyping. Hence, as a limitation of the study, the physical output during an ice hockey match may be affected by various factors including tactical role, playing position, opponent strength, and outcome of the match as has been documented in part in ice hockey as well as in other team sports, introducing variability which may partly affect our results (Gamble et al., 2022; Gregson et al., 2010). For example, large distinctions in activity profile were observed among defensemen and forwards in the present study, especially for high‐intensity skating distance per min. However, forwards also exhibited trends toward higher protein abundance of several proteins related to ion and metabolic regulation and it is unknown whether this reflects potential adaptations to their position‐specific high‐intensity game activity pattern, allowing them the capacity to perform more high‐intensity skating or whether this is random variability partly driving the general associations. Moreover, the sample size, specifically of the defensemen position was relatively low, and more experiments in larger groups of athletes are therefore warranted. On the contrary, clear strengths of the study are the involvement of elite ice hockey athletes accustomed to years of intense ice hockey‐specific training and the application of a high time resolution match analyzing system providing a detailed evaluation of physical game performance. Finally, the addition of supportive data of physical capacity within the endurance, high intensity, and strength performance domain in combination with broad‐spectrum characterization of skeletal muscle phenotype is an important asset of the study.

In conclusion, some skeletal muscle proteins playing a central role in ion and metabolic regulation seem to be associated with ice hockey total and high‐intensity skating performance, with the most consistent findings for NAKα_2_ and K_ATP_ channels, as well as CS enzyme activity within forwards. On the contrary, no associations were found for type IIa myosin heavy chain distribution, whereas several additional associations were less clear when assessed within distinct positions, suggesting the need for more comprehensive follow‐up experiments to further delineate these interrelations.

## AUTHOR CONTRIBUTIONS

JFVL, HT, and MM conceived and designed the research study. JFVL, HT, JP, BF, MBR, JLO, PK, KO, LN, and MM performed the experiments. HT and MT performed the muscle analysis. JFVL and HT analyzed the data. JFVL, HT, KO, LN, and MM interpreted the results of the experiments. JFVL and HT prepared the figures and tables. JFVL, HT, and MM drafted the manuscript. All authors edited and revised the manuscript and approved the final version.

## FUNDING INFORMATION

The study was supported by a grant for The PRoKIT network from The Novo Nordisk Foundation to Team Danmark (grant number NNF.22SA0078293).

## CONFLICT OF INTEREST STATEMENT

The authors have no relevant financial or non‐financial interests to disclose.

## ETHICS STATEMENT

We declare that the present work has been conducted with appropriate ethical approval and informed written consent from the participants.

## Supporting information


Appendix S1.

## Data Availability

Data sets generated during and/or analyzed during the current study are available from the corresponding author on reasonable request.
