# Supplementary material for: Associations between skeletal muscle phenotype, positional role, and on‐ice performance in elite male ice hockey players
Source: Physiol Rep. 2024 Nov 10;12(21):e70081. doi: 10.14814/phy2.70081 (PMC11551070; doi:10.14814/phy2.70081)
Supplement: Supplementary file 1 — Appendix S1. [file PHY2-12-e70081-s001.pptx]

## Slide 1
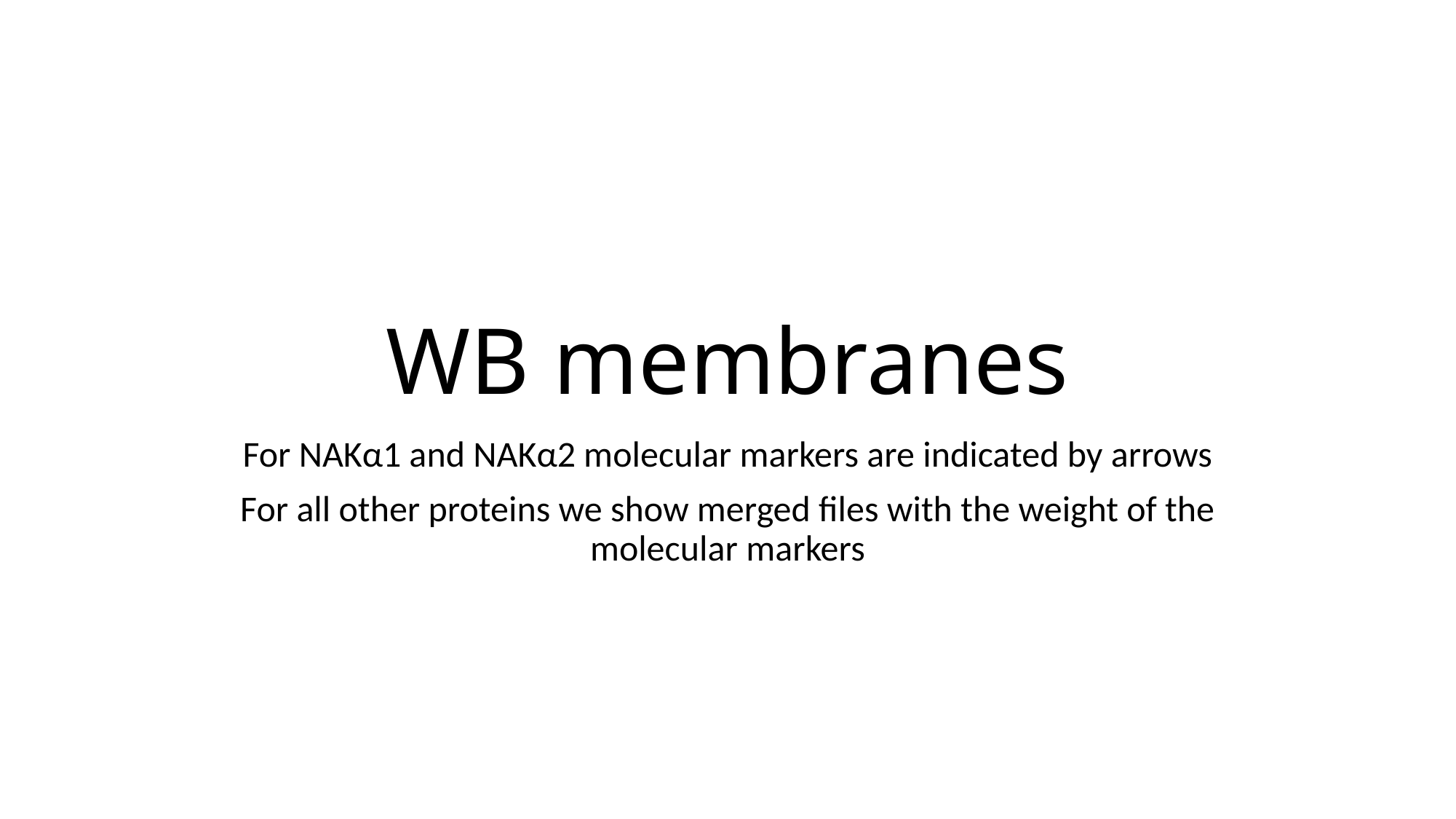

# WB membranes
For NAKα1 and NAKα2 molecular markers are indicated by arrows
For all other proteins we show merged files with the weight of the molecular markers

## Slide 2
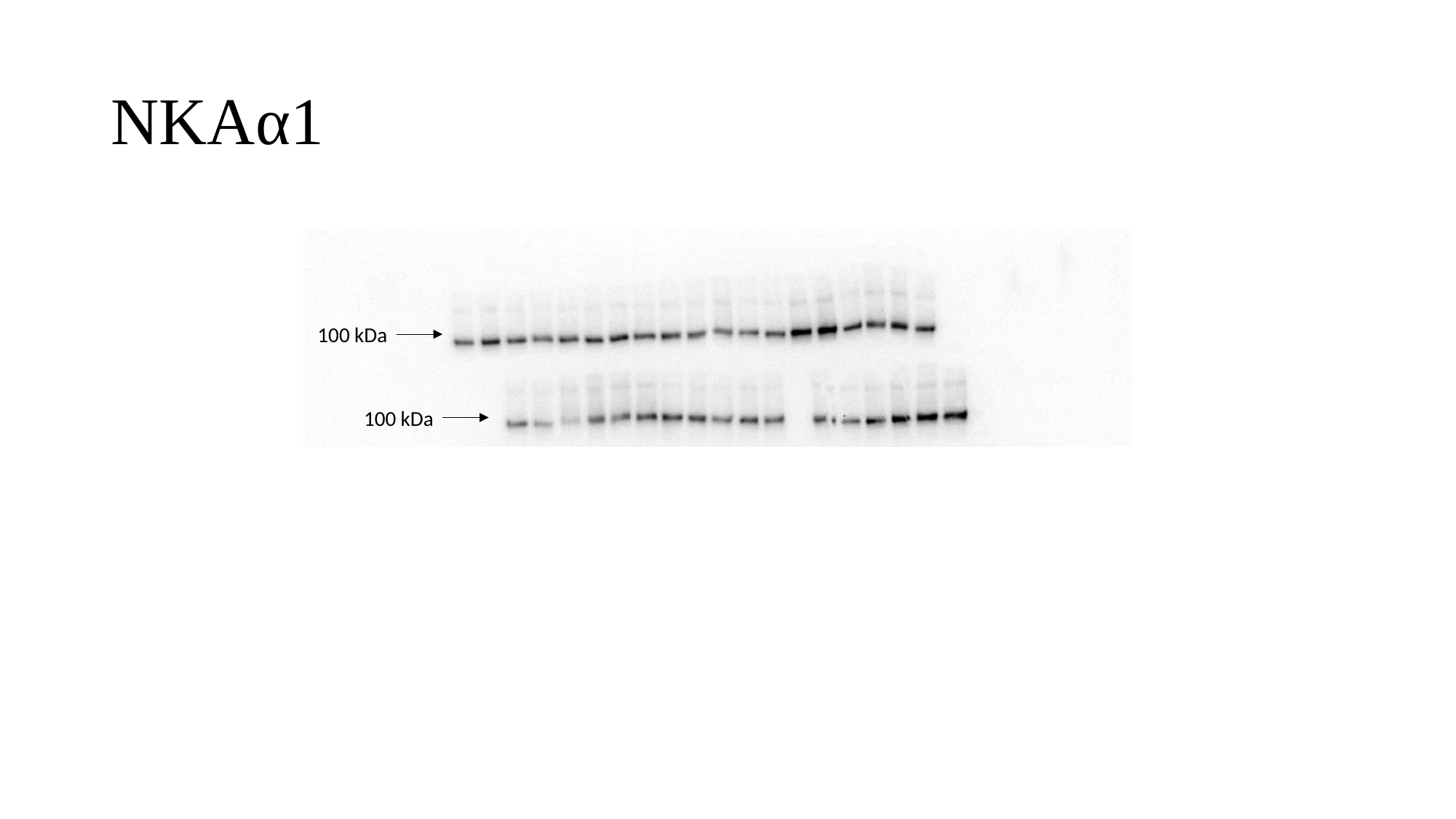

# NKAα1
100 kDa
100 kDa

## Slide 3
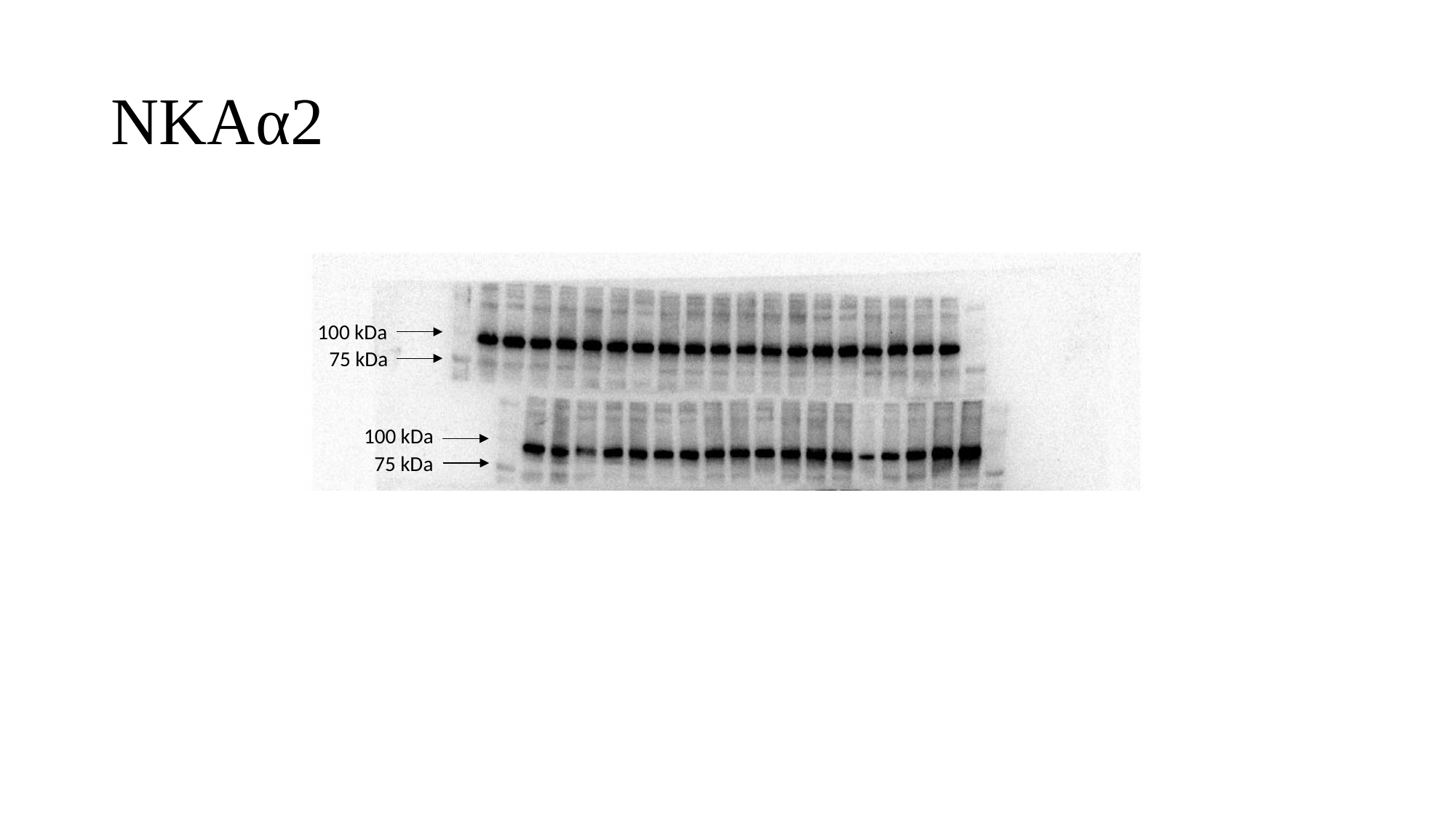

# NKAα2
100 kDa
75 kDa
100 kDa
75 kDa

## Slide 4
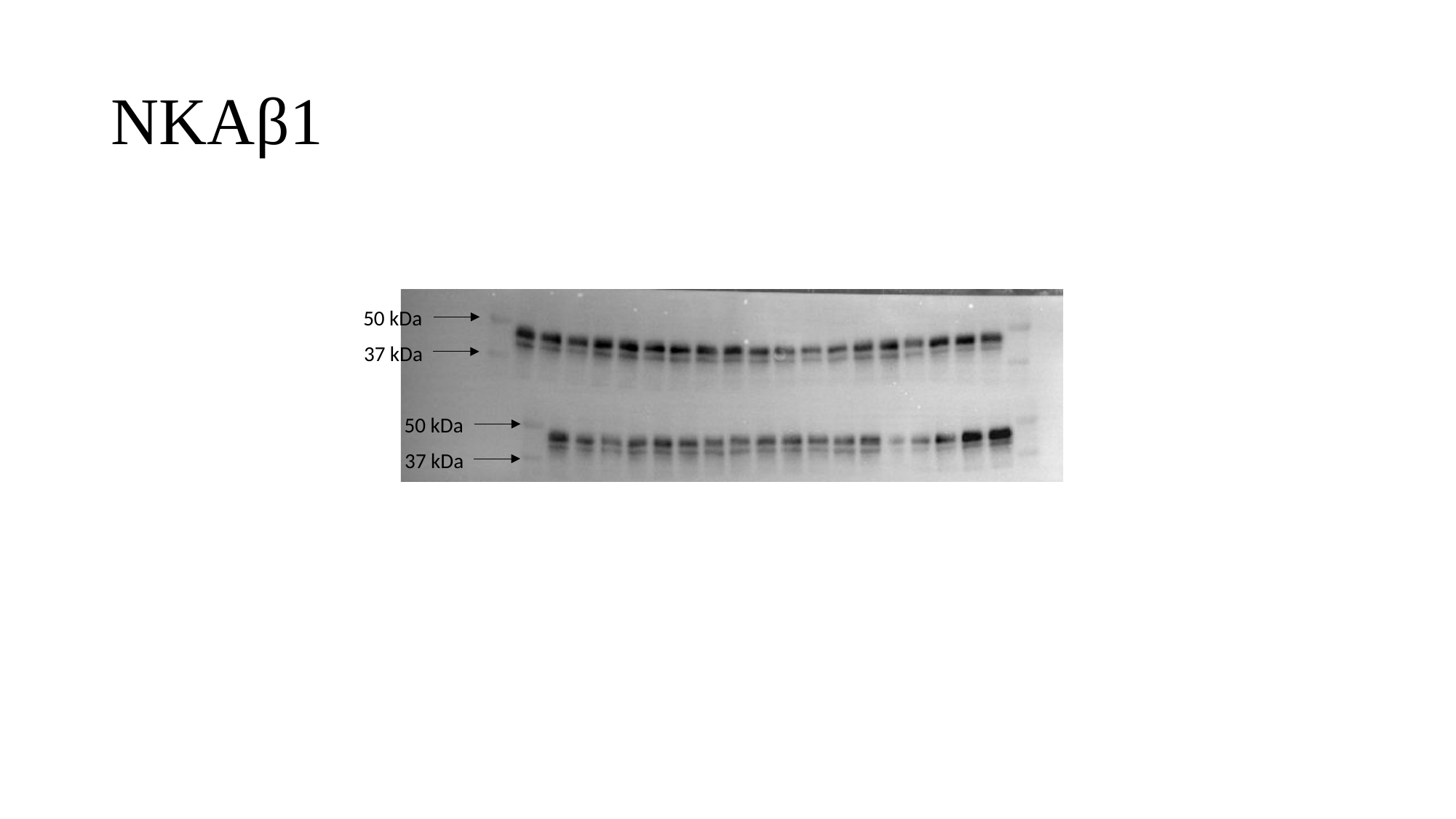

# NKAβ1
50 kDa
37 kDa
50 kDa
37 kDa

## Slide 5
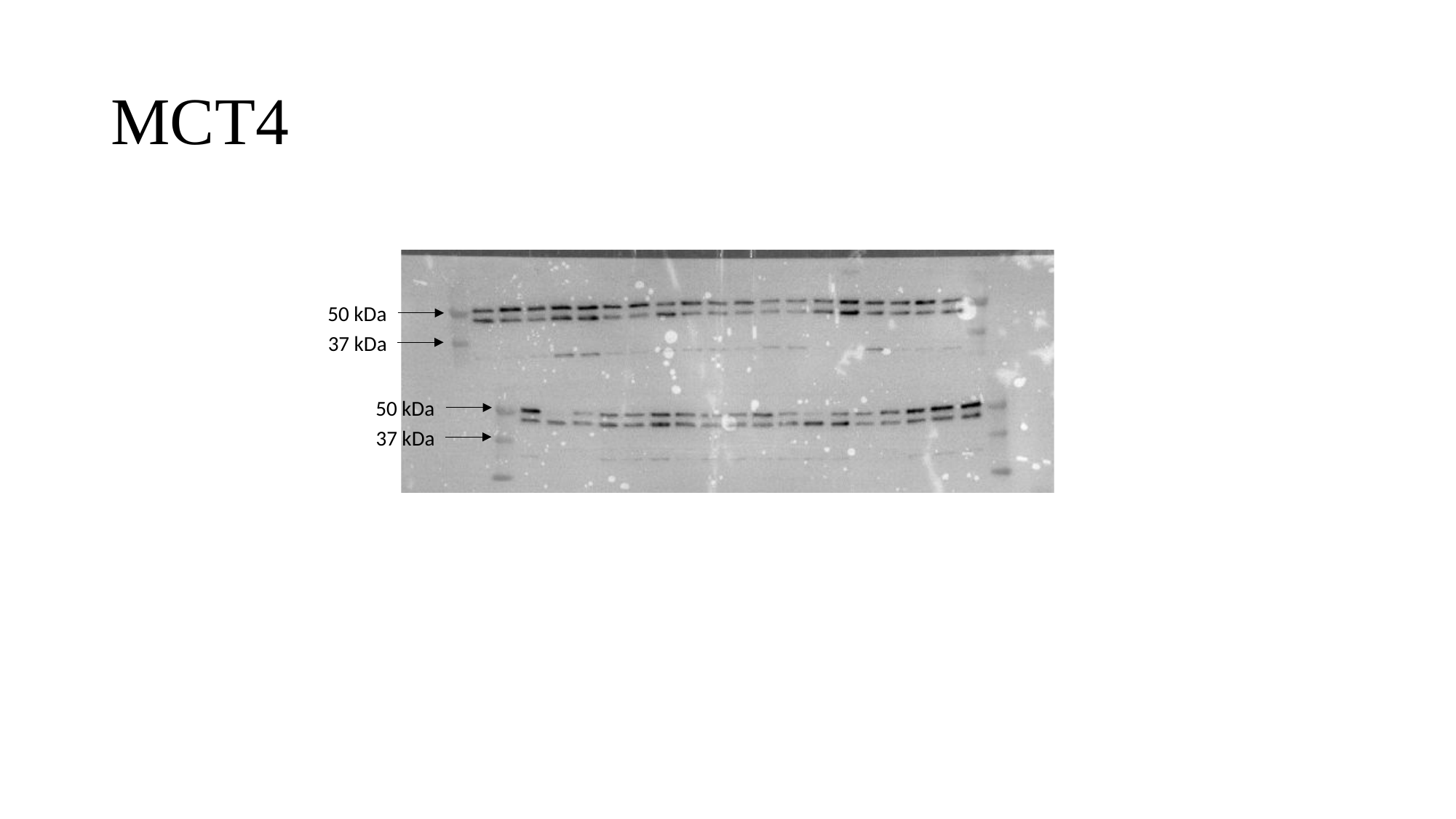

# MCT4
50 kDa
37 kDa
50 kDa
37 kDa

## Slide 6
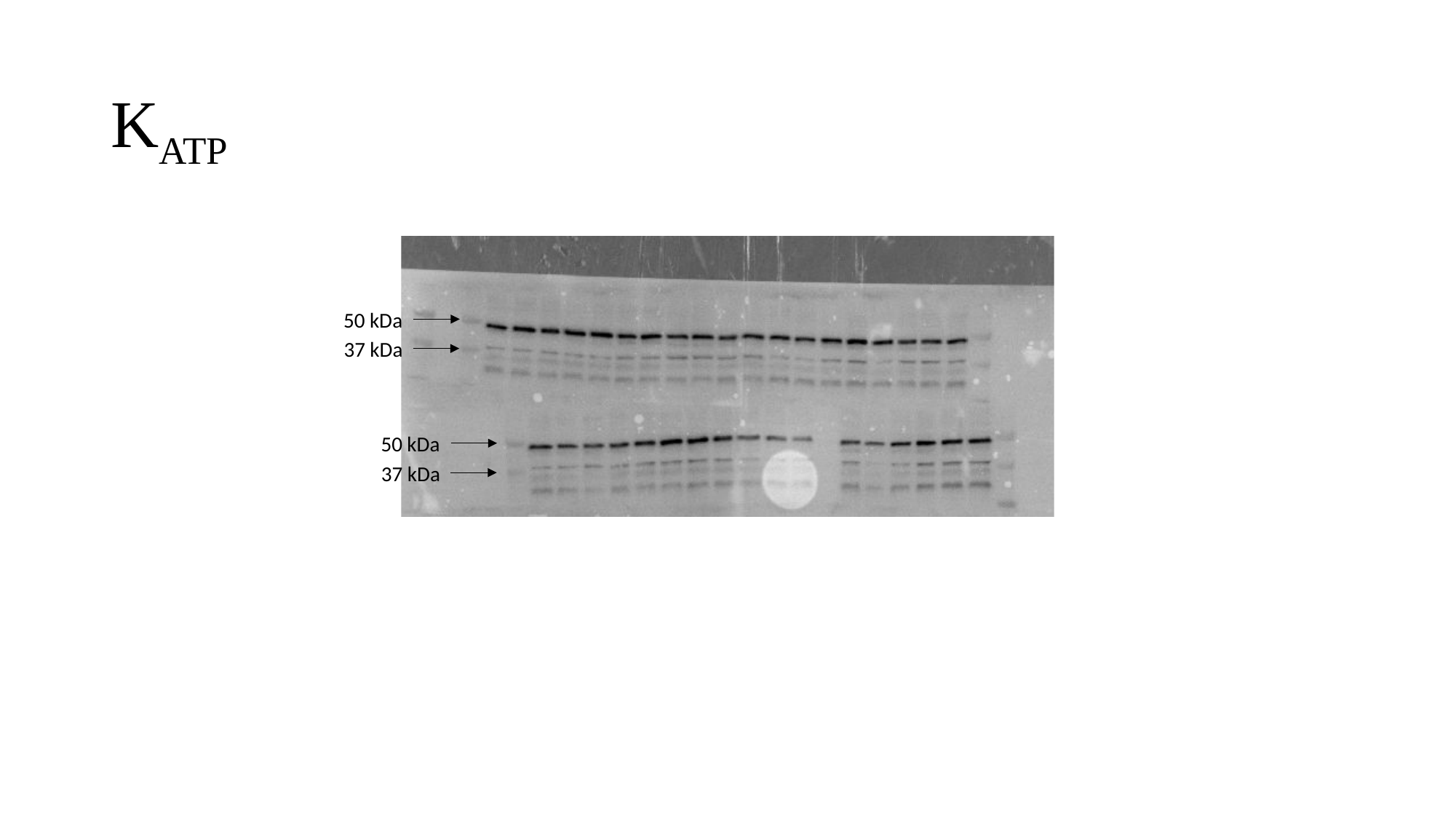

# KATP
50 kDa
37 kDa
50 kDa
37 kDa

## Slide 7
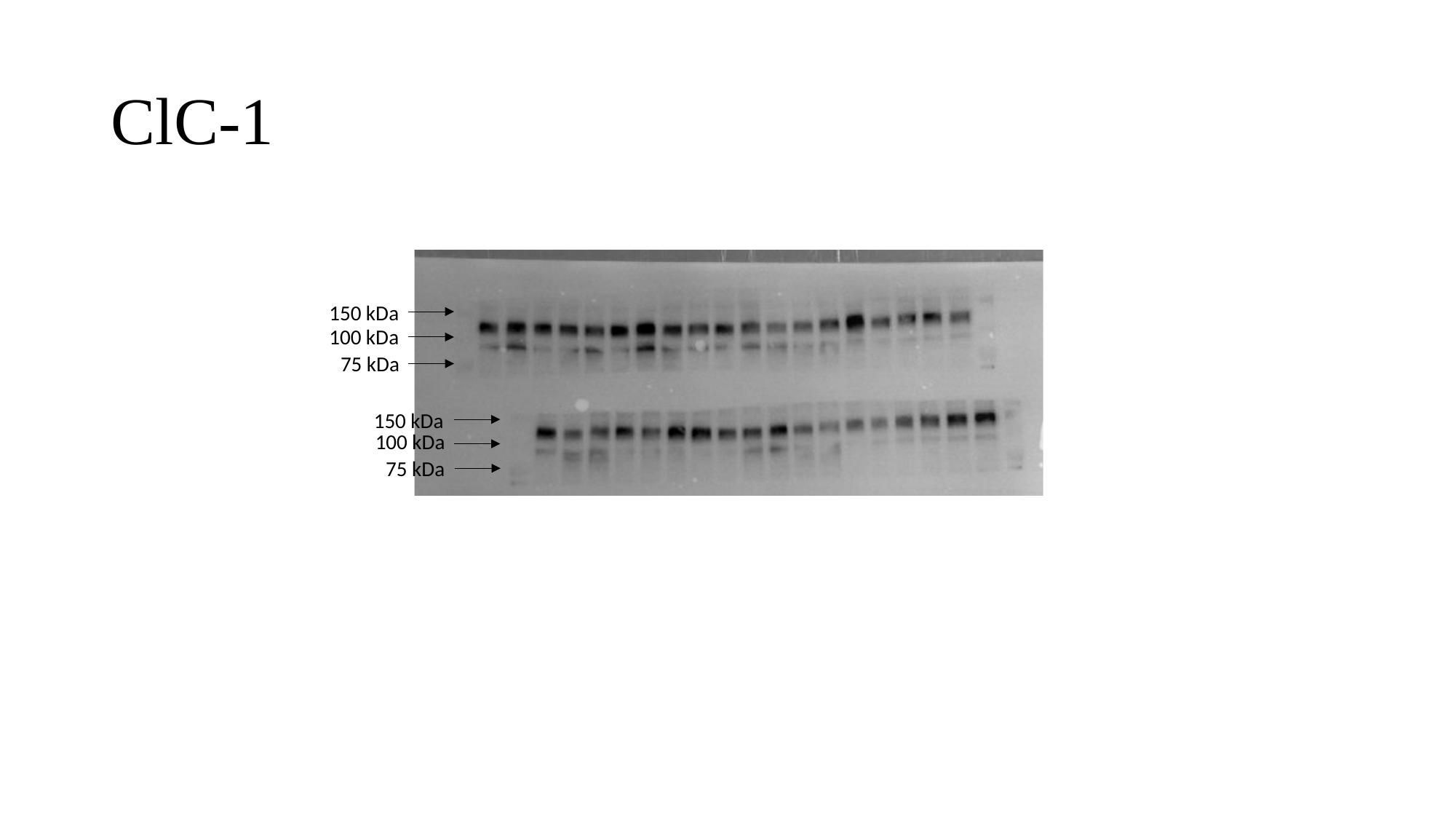

# ClC-1
150 kDa
100 kDa
75 kDa
150 kDa
100 kDa
75 kDa

## Slide 8
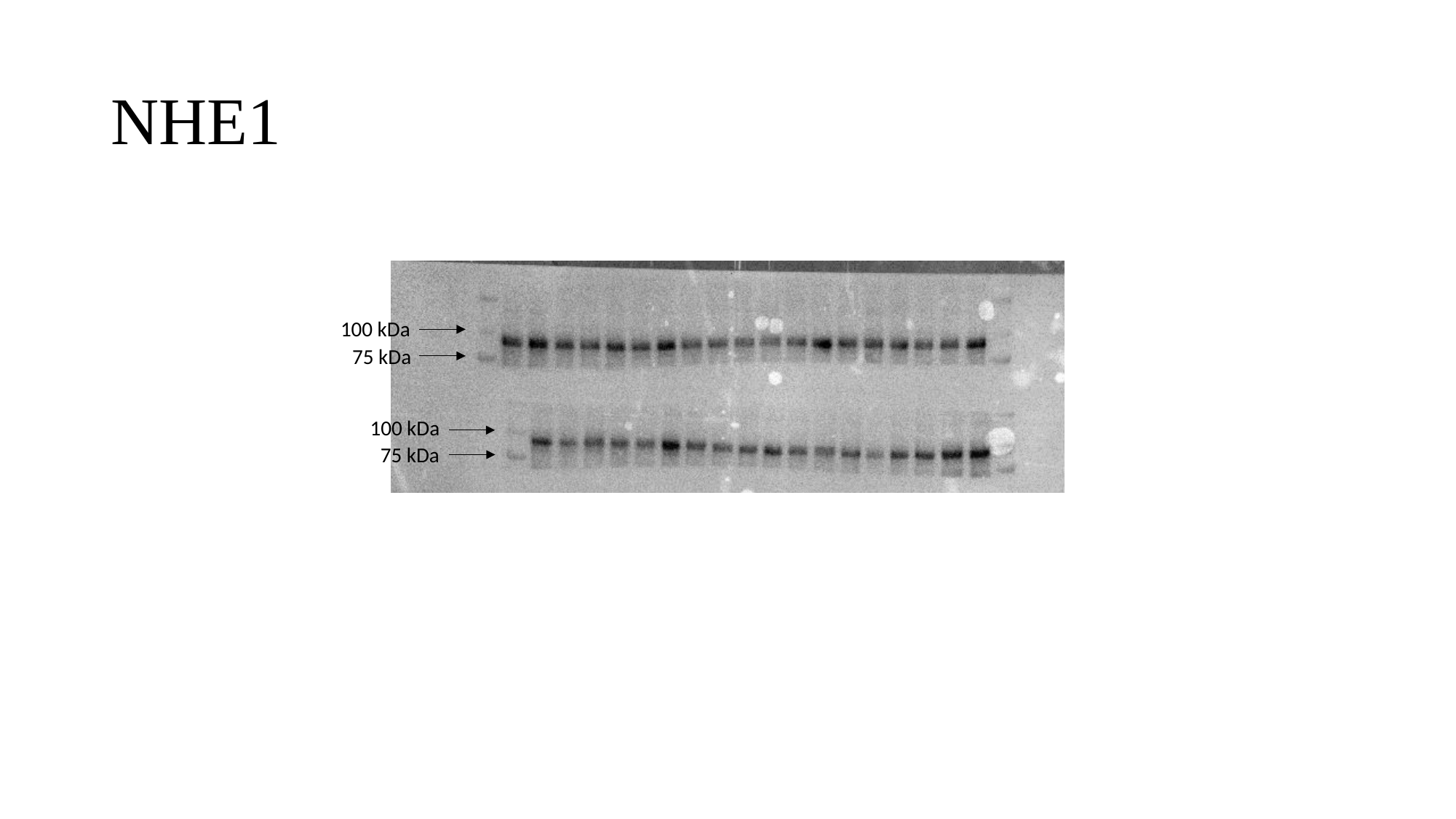

# NHE1
100 kDa
75 kDa
100 kDa
75 kDa

## Slide 9
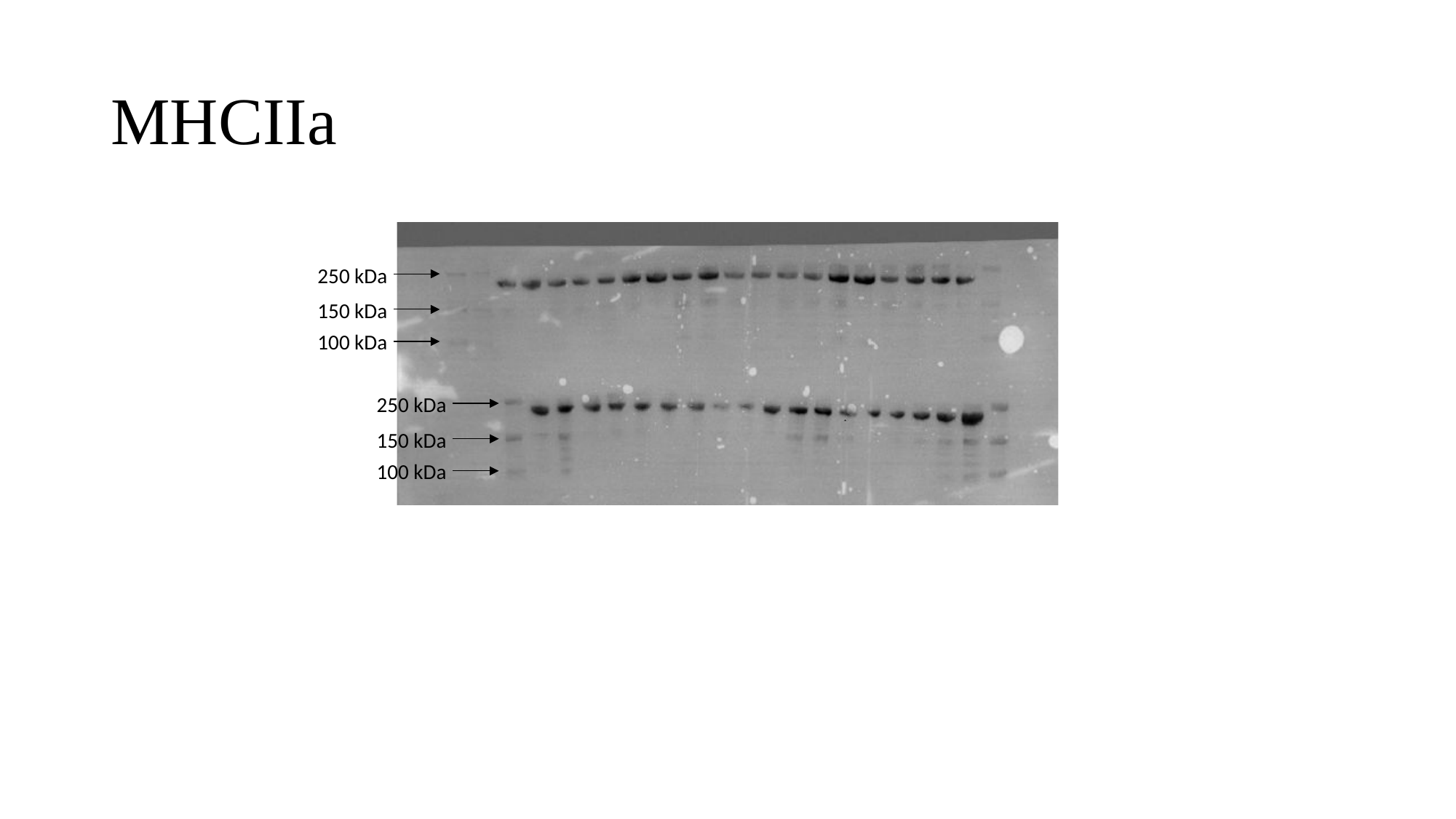

# MHCIIa
250 kDa
150 kDa
100 kDa
250 kDa
150 kDa
100 kDa
